# Positive Effect of Higher Adult Body Mass Index on Overall Survival of Digestive System Cancers Except Pancreatic Cancer: A Systematic Review and Meta-Analysis

**DOI:** 10.1155/2017/1049602

**Published:** 2017-08-29

**Authors:** Jie Han, Yumei Zhou, Yuxiu Zheng, Miaomiao Wang, Jianfeng Cui, Pengxiang Chen, Jinming Yu

**Affiliations:** ^1^Department of Radiation Oncology, Shandong Cancer Hospital and Institute Affiliated to Shandong University, Jinan 250000, China; ^2^Department of Oncology, People's Hospital of Rizhao, Rizhao 2768, China; ^3^Department of Hematology, Qilu Hospital of Shandong University, Jinan 250000, China; ^4^Department of Urology, Qilu Hospital of Shandong University, Jinan 250000, China; ^5^Department of Radiation Oncology, Qilu Hospital of Shandong University, Jinan 250000, China

## Abstract

High body mass index (BMI) has been inconsistently associated with overall survival (OS) of digestive system cancers (DSCs). This meta-analysis was conducted to investigate whether high BMI was associated with DSCs prognosis. 34 studies were accepted, with a total of 23,946 DSC cases. Hazard ratios (HRs) with 95% confidence intervals (95% CIs) for OS in BMI categories from individual studies were extracted and pooled by random-effect model. The overall HR of DSCs except pancreatic cancer for OS of adult overweight cases was 0.76 (95% CI = 0.67–0.85). DSC individuals except pancreatic cancer with adult obesity were at decreased risk for OS (HR = 0.85, 95% CI = 0.72–0.98). Among DSC patients except pancreatic cancer, the overall HR for the highest versus the lowest BMI category was 0.82 (95% CI = 0.71–0.92). Additionally, comparing the highest and lowest BMI categories, the combined HR of pancreatic cancer was 1.22 (95% CI = 1.01–1.43). Our meta-analysis suggested an increased OS among adult overweight and obese DSC survivors except pancreatic cancer. Overweight and obesity in adulthood may be important prognostic factors that indicate an increased survival from DSC patients except pancreatic cancer.

## 1. Introduction

Digestive system cancers (DSCs) are the most common malignancies, accounting for nearly 30% of all cancers [1]. Approximately 350,000 new DSC cases, including oral cavity and oropharynx, are expected with 160,000 estimated deaths in the United States every year [1]. Colorectal cancer, gastric cancer, esophageal cancer, and pancreatic cancer, belonging to DSC, are for high morbidity and mortality rate [2]. The World Cancer Research Fund recommends that cancer patients should keep their weight within normal body mass index (BMI). Excess body weight, whether in overweight (defined as BMI of 25 to 29.9 kg/m^2^) or obese (BMI ≥ 30 kg/m^2^) people, is recognized as an important risk factor for several common cancers [3, 4]. However, studies, focusing on the relationship between BMI and mortality among DSC patients, have reported inconsistent results [5, 6]. On the one hand, some research revealed that higher adult or diagnosis BMI was associated with lower overall mortality [7–9]. Whereas on the other hand, some studies, investigating both adult and diagnosis BMI among DSC patients, suggested that there is no significant relationship between BMI and OS [10–13]. Nevertheless, most results were not statistically significant. Recently, Shi et al. have made a meta-analysis of the association between BMI and OS of pancreatic cancer. Their analysis showed that obesity in adulthood shortened OS of pancreatic cancer patients (HR = 1.29, 95% CI = 1.17–1.41) [14]. This result about pancreatic cancer and BMI was opposed to research on other DSCs, so the analyses of pancreatic cancer and other DSCs should be separated. Moreover, DSC survivors need recommendations on lifestyle factors, and BMI is an important research question to enhance the survival and life quality of particular patients.

We carried out a meta-analysis of published articles to clarify the association between BMI and survival among DSC patients. Moreover, we summarized the evidence on adult and diagnosis BMI and analyzed the highest versus the lowest category of BMI and OS of DSC patients.

## 2. Methods

### 2.1. Search Strategy

Two authors performed the search independently in PubMed, Embase, and the Cochrane Library from its earliest available date to January 20, 2017. The following keywords were used: digestive system, esophagus, esophageal, stomach, gastric, colon, rectum, colorectum, liver, hepatic, gallbladder, pancreas, pancreatic, tongue, oropharynx, cancer, tumor, neoplasm, mortality, survival, BMI, and body mass index. Boolean logic words were jointly used to combine the key words. Potentially relevant articles were investigated seriously by two authors. We also checked the references of retrieved articles for further relevant studies. Disagreements were resolved by group discussion.

### 2.2. Selection of Studies

Studies were considered eligible if they satisfied all the following items: (1) comparing OS of DSC patients with different BMI ranges, containing comparison and referent BMI group; (2) presenting an association estimate with 95% CI or survival curve; (3) only full texts written in English were included. If different articles reported the same study, we only included the publication with the largest size.

### 2.3. Quality Assessment

Two authors independently drew up the evaluation program and assessed full texts included. The Newcastle Ottawa Scale (NOS) was used for methodological quality, which was recommended by the Cochrane Nonrandomized Studies Methods Working Group [15]. This quality evaluation method assessed studies in three dimensions: selection (4 stars), comparability (2 stars), and outcome or exposure (3 stars), with a total score of 9 stars. Studies that scored ≥ 7 were considered as adequately conducted. A third author was involved to solve the disagreement in the scores by consensus.

### 2.4. Data Extraction

Three authors extracted information independently, and disagreements were resolved by consensus. The following data were extracted from each eligible study: first author, year of publication, country where the study conducted, study type, study period, cancer type, cancer site, number of cases, BMI category, both univariate HR (95% CI) and multivariate HR (95% CI) from each BMI category, and covariates list. If data above had not been referred in original articles, items were deemed as “NA.” Engauge Digitizer software was used to extract relevant data and calculate the HR (95% CI) from Kaplan-Meier survival curves [16].

### 2.5. Statistical Analysis

Our analysis evaluated the reported OS of DSC cases with different BMI categories. The highest and lowest BMI group were compared to reveal the mortality difference of DSC. Multivariate HRs were commonly adopted to estimate included studies. Univariate HRs were used instead if multivariate HRs were not available. The pooled HR with 95% CI was estimated by random-effect model. Study-specific study size and 95% CI was showed by forming forest plots. For dose-response evaluation, midpoint of comparison and referent BMI group was used to quantitatively calculate the OS change. If the BMI category was open-ended, midpoints were estimated using the width of the adjacent close-ended category [17]. Subgroup analysis of highest versus lowest BMI category and OS was conducted by study type (retrospective and prospective study), geographic area (North America and other regions), number of cases (≤500 and >500), adjusting for covariate (yes and no), adjusting for weight loss (yes and no), adjusting for tumor grade (yes and no), and cancer source (oropharynx, esophagus, stomach, colorectum, and pancreas). Sensitivity analysis was carried out to examine the impact of single study. Every time one study was excluded, and the rest was analyzed to evaluate whether single study affects results significantly. Heterogeneity was assessed by *Q* and *I*^2^ statistics. A pooled HR > 1 revealed that comparison BMI group had worse prognosis than referent group for DSC patients. On the other hand, a pooled HR < 1 suggested comparison BMI group predicted a more favorable survival. When the 95% CI of HR did not overlap 1, the result was regarded as statistically significant. Begg funnel plots and Egger regression asymmetry test were used to evaluate publication bias. All *P* values were 2 sides. *P* < 0.05 was considered as statistically significant. All analyses were performed using STATA version 12.0 software (Stata Corporation, College Station, TX). Our research did not need ethical approval or consent as this meta-analysis was a review of published studies.

## 3. Results

### 3.1. Literature Search and Study Characteristics

**Figure 1:**
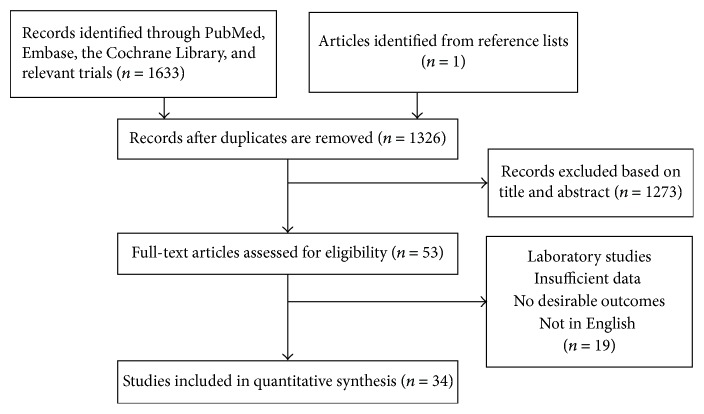
Flowchart of study selection in this meta-analysis. 1634 studies were preretrieved in accordance with the established search strategies. Then 53 studies that may meet the requirements were further screened out through browsing the titles and abstracts. After reading the full texts of 53 studies, 34 eligible studies were finally included in this meta-analysis according to the criteria.

The search strategy identified 1633 articles. Excluding irrelevant articles and duplicates, remaining 35 full texts were assessed for eligibility. Additional one record was identified from reference lists. 36 articles met the inclusion criteria and were assessed for eligibility. Further examination led to exclude two studies (Figure 1). Although some excluded studies provided survival curve, we cannot extract or calculate HR and 95% CI from the article. In addition, the study from Ishizuka et al. had not been finished yet and was presented as a poster [18].

In total, we included 34 studies in meta-analysis. We combined and evaluated 6 kinds of cancers: tongue [19], oropharyngeal [10, 20, 21], esophageal [5–7, 11, 12, 22–28], gastric [8, 13, 29, 30], colorectal [9, 31–33], and pancreatic cancer [34–43]. Four studies referred the OS of both overweight and obese patients in adulthood [9, 22, 32, 33, 44]. Two studies reported the survival of both overweight and obese patients at diagnosis [19, 30]. All of the articles were published between 2005 and 2016; there were 23 prospective studies and 11 retrospective studies. 11 studies were from the North American, and the remaining 13 studies were from other regions. 19 included studies contained more than 500 cases, and the remaining 15 had less than 500 patients. The referent group from more than half of the studies was normal BMI category. Most studies provided multivariate HR and 95% CI. These results were adjusted by age, gender, race, smoking, diabetes, tumor stage, lymph node metastasis, treatment, and other covariates (Table 1). All studies that scored ≥ 7 according to NOS were considered as adequately conducted.

### 3.2. Overweight and OS of DSC Except Pancreatic Cancer

**Figure 2:**
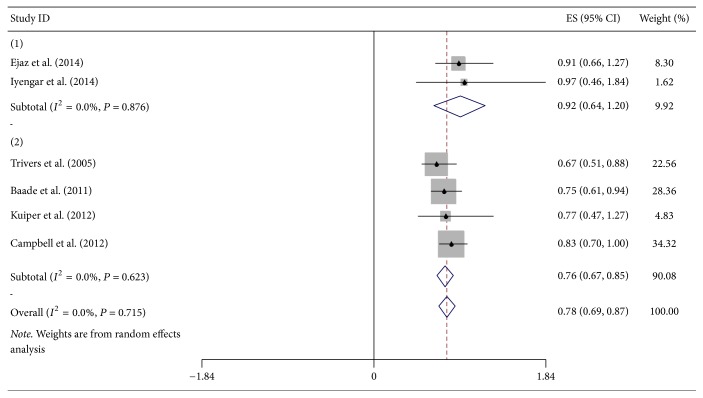
Forest plot showed hazard ratios (HRs) and 95% CIs for overweight and overall survival of DSC except pancreatic cancer. HRs are for BMI at diagnosis and in adulthood. ((1) at diagnosis, (2) in adulthood).

Association between overweight and OS of DSC, excluding pancreatic cancer, was presented in six studies (five prospective and one retrospective study) (Figure 2). The pooled HR for overweight at diagnosis of DSC patients except pancreatic cancer was 0.92 (95% CI = 0.64–1.20). Additionally, the combined result informed that adult overweight was significantly associated with OS of DSC patients except pancreatic cancer (HR = 0.76, 95% CI = 0.67–0.85). In total, overall HR of six studies was 0.78 (95% CI = 0.69–0.87). All above analyses were with no significant heterogeneity; all *I*^2^ = 0% and *P*_heterogeneity_ = 0.876, 0.623, and 0.715, respectively. The study of Iyengar et al. just contributed to 1.62% of overall HR, while total weight of three prospective studies was 85.24%. Compared with normal BMI, increased adult BMI was related to lower risk of death with 9% for every 5-unit increment.

### 3.3. Obesity and OS of DSC Except Pancreatic Cancer

**Figure 3:**
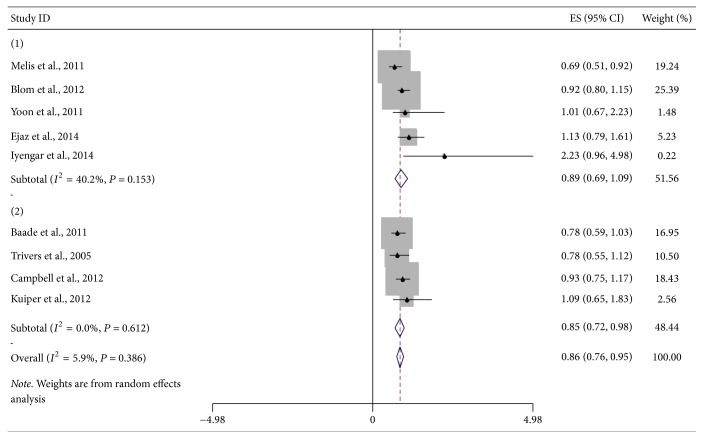
Forest plot showed hazard ratios (HRs) and 95% CIs for obesity and overall survival of DSC except pancreatic cancer. HRs are for BMI at diagnosis and in adulthood. ((1) at diagnosis, (2) in adulthood).

Nine studies (eight prospective and one retrospective study) were included in the analysis of obesity and survival of DSC patients except pancreatic cancer (Figure 3). Pooled HR of five studies for obesity at diagnosis time was 0.89 (95% CI = 0.69–1.09). No significant heterogeneity was found (*I*^2^ = 40.2%, *P*_heterogeneity_ = 0.153). Meta-analysis of four prospective studies on the association of adult obesity and OS of DSC participants revealed that pooled HR was 0.85 (95% CI = 0.72–0.98), without obvious heterogeneity (*I*^2^ = 0%, *P*_heterogeneity_ = 0.612). Combined HR of all nine studies was 0.86 (95% CI = 0.76–0.85, *I*^2^ = 5.9%, *P*_heterogeneity_ = 0.386). With every 5-unit of BMI increased in adulthood, risk of death was reduced by 3%.

### 3.4. Highest versus Lowest BMI and OS of DSC Except Pancreatic Cancer

**Figure 4:**
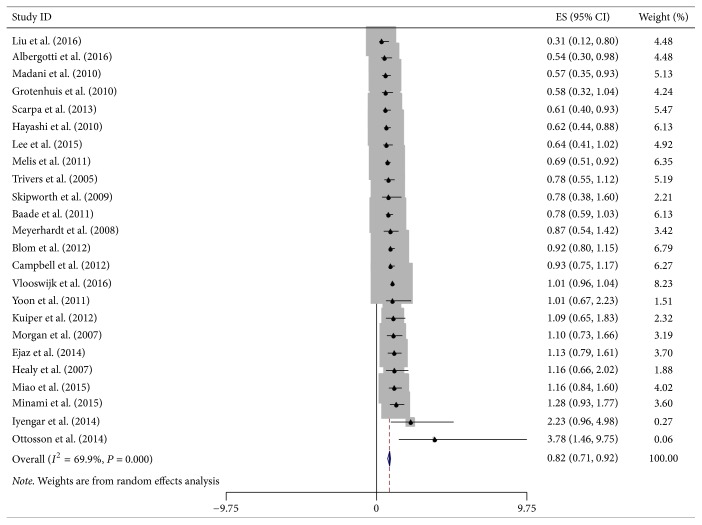
Forest plot showed hazard ratios (HRs) and 95% CIs for the highest versus lowest BMI category and overall survival of DSC except pancreatic cancer.

Twenty-four studies on highest versus lowest BMI and mortality of DSC patients except pancreatic cancer were combined and analyzed (Figure 4). Highest and lowest BMI from all studies, both at diagnosis and in adulthood, were included in this analysis. Compared with lowest BMI category, DSC patients except pancreatic cancer with highest BMI survived longer with an 18% lower risk of death (HR = 0.82, 95% CI = 0.71–0.92), with moderate heterogeneity (*I*^2^ = 69.9%, *P*_heterogeneity_ < 0.001).

### 3.5. Site-Specific Risk Analysis

**Figure 5:**
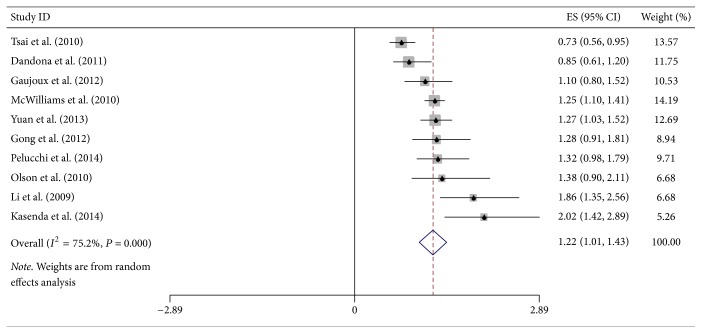
Forest plot showed hazard ratios (HRs) and 95% CIs for the highest versus lowest BMI category and overall survival of pancreatic cancer.

**Figure 6:**
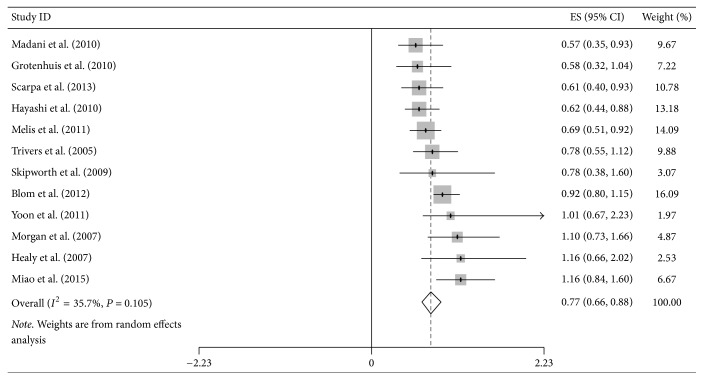
Forest plot showed hazard ratios (HRs) and 95% CIs for the highest versus lowest BMI category and overall survival of esophageal cancer.

**Figure 7:**
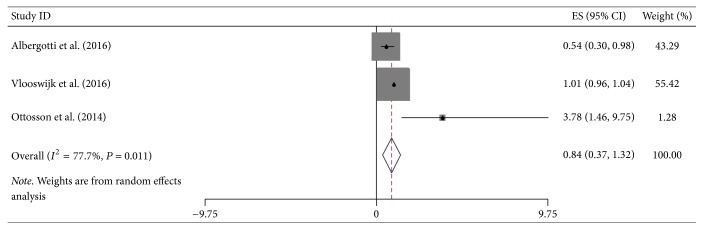
Forest plot showed hazard ratios (HRs) and 95% CIs for the highest versus lowest BMI category and overall survival of oropharyngeal cancer.

**Figure 8:**
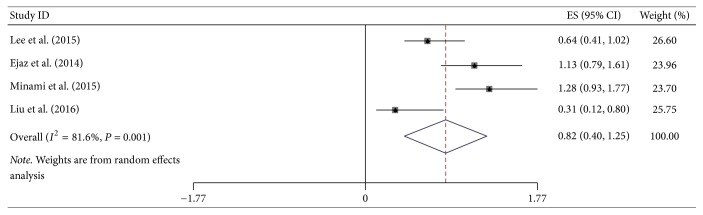
Forest plot showed hazard ratios (HRs) and 95% CIs for the highest versus lowest BMI category and overall survival of gastric cancer.

**Figure 9:**
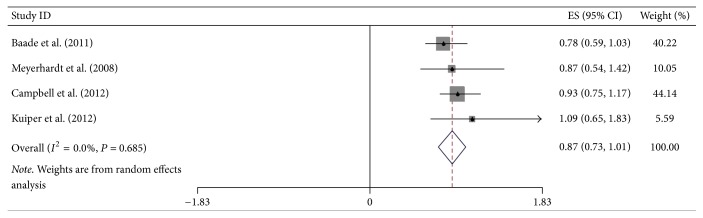
Forest plot showed hazard ratios (HRs) and 95% CIs for the highest versus lowest BMI category and overall survival of colorectal cancer.

Additional site-specific tumor risk estimate was also conducted in this meta-analysis. We combined and analyzed ten studies on cancers of pancreas, three of oropharynx, twelve of esophagus, four of stomach, and four of colorectum, respectively. Pooled HR for highest versus lowest BMI category of pancreatic cancer was 1.22 (95% CI = 1.01–1.43), but the heterogeneity was high (*I*^2^ = 75.2%, *P*_heterogeneity_ < 0.001) (Figure 5). Regarding the highest versus lowest BMI category, there was significant association for the OS of esophageal cancer survivors (HR = 0.77, 95% CI = 0.66–0.89) (Figure 6). However, no significant association was found in the subgroup analysis of some site-specific tumors, including oropharynx (HR = 0.84, 95% CI = 0.37–1.32), stomach (HR = 0.82, 95% CI = 0.40–1.25) and colorectum (HR = 0.87, 95% CI = 0.71–1.01) (Figures 7, 8, and 9).

### 3.6. Subgroup Analysis

In subgroup analysis, comparing with the lowest BMI category, the highest category had a statistically significant effect on OS of DSC patients except pancreatic cancer in both North America and non-North America group, with the HR of 0.77 (95% CI = 0.65–0.89) and 0.84 (95% CI = 0.70–0.99) (Table 2). Moreover, both groups of sample size were statistically significant. Regarding the highest versus lowest BMI category and OS of DSC patients except pancreatic cancer, there was no significant association for retrospective studies and these adjusted for weight loss and tumor grade. Additionally, subgroup analysis of studies, which were not adjusted for any covariates, did not show significant association of OS of DSC except pancreatic cancer with highest versus lowest BMI comparison.

### 3.7. Sensitivity Analysis and Publication Bias

**Figure 10:**
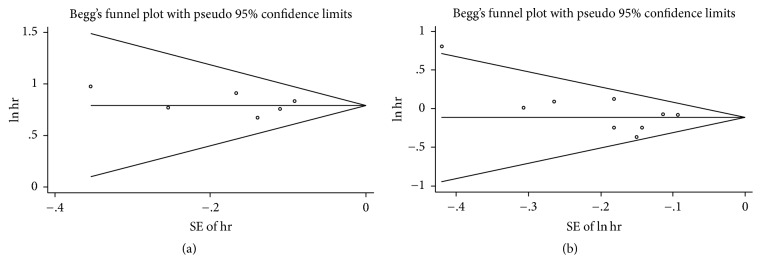
Begg funnel plot test for higher BMI and overall survival of DSC except pancreatic cancer. ((a) overweight, (b) obesity).

**Figure 11:**
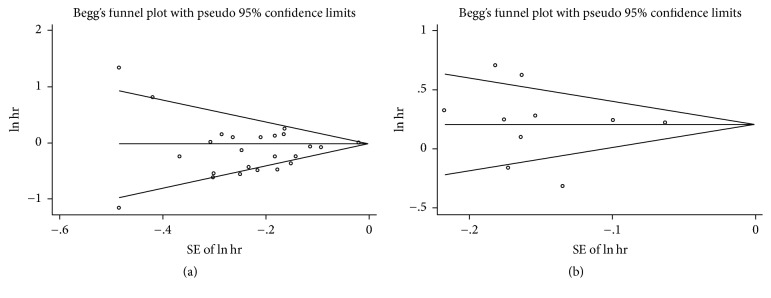
Begg funnel plot test for the highest versus lowest BMI and overall survival of DSC. ((a) DSC except pancreatic cancer, (b) pancreatic cancer).

**Figure 12:**
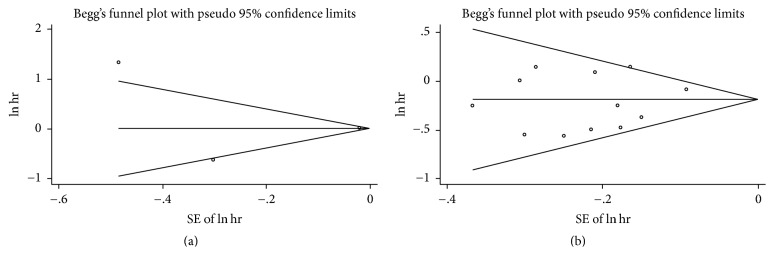
Begg funnel plot test for the highest versus lowest BMI and overall survival of DSC. ((a) oropharyngeal cancer, (b) esophageal cancer).

**Figure 13:**
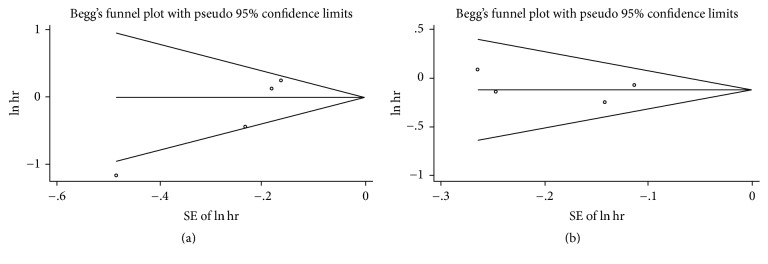
Begg funnel plot test for the highest versus lowest BMI and overall survival of DSC. ((a) gastric cancer, (b) colorectal cancer).

In sensitivity analysis, we excluded one study every turn and analyzed the rest of the articles. No significant change of pooled HR and 95% CI occurred when every single study was ignored. In publication bias, we used Begg funnel plot and Egger regression test to assess bias. The funnel plot for OS of DSC patients except pancreatic cancer and overweight (Begg test *P* = 0.467) or obesity (Begg test *P* = 0.329) showed no asymmetry (Figure 10). Begg test for highest versus lowest BMI category and mortality of pancreatic cancer (*P* = 0.867) or other DSCs (*P* = 0.086) failed to reveal any significant publication bias (Figures 11, 12, and 13). Egger regression test for all groups also suggested no obvious publication bias.

## 4. Discussion

Overweight and obesity account for approximately 20% of all cancer patients, including esophageal adenocarcinoma, colorectal cancer, and pancreatic cancer (RR range from 1.07 to 1.52, for male cases) [44–46]. Apart from cancers, obesity was observed to be related to cardiovascular disease, chronic kidney disease, sleeping disorder, and type 2 diabetes.

The relationship between BMI and DSCs has been discussed for decades. Three published meta-analyses had evaluated the association between BMI and survival of particular DSC patients, including esophageal cancer, colorectal cancer, and pancreatic cancer [14, 47–49]. Zhang et al. estimated the highest versus lowest BMI category and OS of esophageal cancer survivors. Their study found that high BMI could significantly improve OS (HR = 0.78, 95% CI = 0.71–0.85) [49]. However, a study of 1324 esophageal cancer participants, which was conducted in 2014, revealed that high BMI is not associated with increased overall morbidity after esophagectomy [28]. Additionally, Zhang et al. just analyzed the highest versus lowest BMI category and complications, avoiding considering the time point and accurate BMI category [49]. Another meta-analysis attempted to explicate the question of postdiagnosis BMI and mortality of colorectal cancer cases. Results indicated that overweight individuals had a lower all-cause mortality (HR = 0.79, 95% CI = 0.71–0.88). For obese subjects, the risk of mortality was reduced with borderline significance (HR = 0.88, 95% CI = 0.77–1.00) [48]. Postdiagnosis BMI may contain BMI after diagnosis, during treatment, and after treatment. Moreover, BMI in adulthood was also an important prognostic factor of mortality of colorectal cancer survivors. Shi et al. had made the latest meta-analysis of BMI and OS of pancreatic cancer in April of 2016. Results suggested that adult obesity of pancreatic cancer cases may shorten OS (HR = 1.29, 95% CI = 1.17–1.41), while obesity at diagnosis was not associated with the mortality (HR = 1.10, 95% CI = 0.78–1.42) [14].

The mechanism behind the results that adult obesity enhanced the OS of pancreatic cancer had not been revealed thoroughly. Increased insulin resistance, DNA damage, adipokines, and proinflammation may contribute to the survival outcomes [50]. Additionally, we found that the influence of higher BMI on pancreatic cancer was different from other DSCs. The unique structure and function of pancreas and special characteristic of pancreatic cancer may result in the difference.

Prognostic effect of overweight and obesity on DSCs has been searched. However, the role of BMI at diagnosis and in adulthood on the mortality of DSC patients is still unclear, excluding pancreatic cancer with a latest meta-analysis. Thus, we conducted the meta-analysis to identify the prognostic role of BMI on overall survival from DSCs except pancreatic cancer. Additionally, we analyzed the association between the highest BMI versus lowest BMI category and OS of pancreatic cancer to cover the shortage of Shi et al.'s research.

Eighteen included studies were multivariately analyzed and nine studies provided both multivariate and univariate results. In this study, we conducted each analysis using multivariate results as many as possible. When estimating the highest versus lowest BMI category and mortality, we combined all studies to analyze the association. Some studies, which only provided univariate outcomes, were also adapted to achieve more credible pooled results. Subgroup analyses for covariates adjusting, especially for weight loss and tumor grade, were performed as a supplement. Overweight and obesity in adulthood significantly enhanced the OS of DSC patients except pancreatic cancer. But we failed to find significant association between overweight or obesity at diagnosis and OS of DSCs except pancreatic cancer. Pooled analysis of both overweight and obesity revealed positive effect on survival of DSC patients except pancreatic cancer: HR = 0.78 (95% CI = 0.69–0.87) and HR = 0.86 (95% CI = 0.76–0.95), respectively. Excluding pancreatic cancer, higher adult BMI was related to better survival. Considering the limitation of study numbers, we included all 24 studies and estimated the highest versus lowest BMI category and mortality of DSC patients except pancreatic cancer. It was easier to find the relationship of BMI and OS by analyzing the maximum and minimum BMI. The outcome provided the evidence that patients with higher BMI had lower mortality. Moreover, we included 10 studies on pancreatic cancer and OS to conduct further analysis. The results of supplementary study were coincident with the former study of pancreatic cancer patients [14]. The current HR of the highest versus lowest BMI and OS of pancreatic cancer survivors was 1.22 (95% CI = 1.01–1.43). To further investigate the predictive value of BMI and OS of DSC patients, subgroup analysis was conducted to estimate these factors affecting this study. The analysis results of all geographic area and sample size groups showed significant association between higher BMI group and OS of DSC patients except pancreatic cancer, comparing with lowest BMI group. Combined analysis of different study types revealed contrary results, pooled HR of retrospective study was 0.77 (95% CI = 0.53–1.01), and pooled HR of prospective was 0.84 (95% CI = 0.72–0.95). The results of prospective study were more credible than retrospective study, due to the better controllability. When we analyzed studies adjusted for covariates, the association between highest versus lowest BMI category and OS of DSC patients except pancreatic cancer was statistically significant (HR = 0.80, 95% CI = 0.68–0.93). Regarding highest versus lowest BMI category and OS of DSC patients except pancreatic cancer, subgroup analysis of studies adjusted for tumor grade (HR = 0.99, 95% CI = 0.70–1.28) showed no significant association and weight loss (HR = 0.78, 95% CI = 0.52–1.05). Both weight loss and tumor grade were important covariates for the analysis of OS of DSC patients. Most studies included in this meta-analysis were adjusted for tumor stage, but only six articles estimated the effect of tumor grade. Survival of esophageal cancer patients was strongly dictated by tumor stage after neoadjuvant chemotherapy [51]. Both univariate and multivariable analysis revealed that better tumor grade was associated with longer survival in esophageal cancer cases (*P* = 0.007 and *P* = 0.011, resp.) [52]. Loss of weight and loss of skeletal muscle may indicate the progression of cancer disease [53]. Compared with stable BMI, a prediagnostic BMI decrease was associated with poorer prognosis for OS of colorectal cancer patients (adjusted HR = 1.83, 95% CI = 1.43–2.34) [54]. Campbell et al. suggested that postdiagnosis weight loss was a predictor for mortality of colorectal cancer participants, while weight gain was not related to survival [32]. Additionally, pancreatic cancer cases, with higher weight loss at diagnosis or during first-line chemotherapy, had shortened survival [55].

Risk estimate of site-specific tumors from digestive system is necessary and concerned by many scholars. In esophageal cancer survivors, we obtained meaningful result (HR = 0.77, 95% CI = 0.66–0.89), suggesting that highest BMI group had better survival than lowest group. Although higher BMI represented the trend of better overall survival, the significant effect was not found in groups of oropharyngeal, gastric, and colorectal cancers. By a clinical-based cohort and meta-analysis in 2013, Zhang et al. revealed that high BMI could significantly enhance overall survival of esophageal cancer (HR = 0.78, 95% CI = 0.71–0.85), which was consistent with this study [49]. Another prospective study and meta-analysis indicated a decreased all-cause mortality risk among overweight colorectal cancer patients; HRs (95% CI) for overweight and obesity were 0.79 (0.71–0.88) and 0.88 (0.77–1.00), respectively [48]. Additionally, the relationship between oropharyngeal or gastric cancer and higher BMI is unclear, and our study provided the combined results.

The underlying mechanisms of the effect of higher BMI on DSC patients were unclarified and rarely elucidated. Comparing with normal BMI cases, overweight and obese patients had a better nutrition status and potential survival advantages [56]. DSC patients with higher adult BMI had more nutrient and energy stores during treatment. They had larger appetites and higher lipid concentration for preserving energy, fat, and muscle mass. Higher food intake could provide more necessary body elements, promote tissue repair, keep physiological activities, and enhance the immune effect. However, higher BMI also had a higher incidence of complication after treatment. Overweight and obesity in esophageal survivors may induce anastomotic leakage (RR = 1.04, 95% CI = 1.02–1.06), wound infection (RR = 1.03, 95% CI = 1.00–1.05), slow growth of anastomosis, and cardiovascular diseases [6, 11, 12, 23, 47]. Obese cases had higher rate of diabetes mellitus, which may influence the healing of DSC patients after treatment [12]. The mechanisms behind the observation that higher adult BMI is associated with enhanced OS have not been revealed thoroughly. Further study is needed to explain this phenomenon.

To our knowledge, this study is the first meta-analysis estimating the association between BMI and OS of DSC patients. Analysis of a functional system, adjusted risk factors, the relatively large sample size, and the summarized evidence of single study are strengths in our study. However, limitations of our study should be addressed. Most included studies were conducted in developed countries; research in developing countries may be restricted by techniques, devices, therapies, and other factors. Comprehensive and through analysis needs more research information from developing countries. Except pancreatic cancer, we analyzed BMI at two time points: adulthood and diagnosis, and weight loss as a significant prognostic factor was only considered in five included articles [7, 25, 27, 28, 31]. Meyerhardt et al. just described the postoperation BMI of patients; the data may be inaccurate due to rapid weight change around operation. Abdominal obesity may increase the mortality of general population and influence the OS of DSC patients, but we have no information on this independent risk factor [57]. Not all studies provided information about tumor grade, differentiation, lymph node metastasis, diabetes status, and treatment, which were usual covariates for OS of cancer cases. As we know, clinical evidence level of prospective studies is higher than retrospective ones, but this meta-analysis contained both prospective and retrospective studies. Our analysis cannot avoid selection bias, because inclusion of participants depends on survival time. Additionally, a number of severe cases were less than actual proportion in DSC patients.

## 5. Conclusion

This study revealed that overweight and obesity in adulthood increased the OS of DSC patients except pancreatic cancer. However, higher BMI at diagnosis did not show any association with the survival of DSC patients. In total, DSC patients, excluding pancreatic cancer, with higher BMI had better survival than lower BMI. To draw definite recommendations for DSC survivors, further studies are needed to find whether BMI and related clinical factors are potential predictors of mortality of DSC patients.

## Figures and Tables

**Table 1:** Characteristic of relevant studies on BMI and OS of DSC patients included in the meta-analysis.

Study	Country	Study type	Duration	Cancer site	Size	Point of BMI	BMI	Referent BMI	UV-HR (95% CI)	MV-HR (95% CI)	Covariates
Iyengar et al., 2014	USA	Retrospective study	2000–2009	Tongue	155	Before operation	≥30 25.0–29.9	18.5–24.9	1.86 (0.95, 3.63) 1.01 (0.97, 1.03)	2.23 (0.96, 4.98) 0.97 (0.46, 1.84)	Age, race, smoking, diabetes, T stage, tumor grade, invasion, lymph node metastasis, and postoperative radiation

Ottosson et al., 2014	Sweden	Retrospective study	1998–2006	Oropharynx	203	Before radiotherapy	>25	<20 20–25	3.31 (1.4, 7.83) 3.07 (1.74, 5.44)	3.78 (1.46, 9.75) 2.57 (1.43, 4.62)	Age, sex, stage, RT schedule, and surgery

Vlooswijk et al., 2016	Netherlands	Retrospective study	2008–2012	Oropharynx	276	Before radiotherapy	≥25	≤25	1.01 (0.96, 1.04)	NA	NA

Albergotti et al., 2016	USA	Retrospective study	2006–2014	Oropharynx	579	Before treatment	≥25	<25	0.49 (0.28, 0.87)	0.54 (0.3, 0.98)	Age, sex, smoking, race, stage, and drinking

Trivers et al., 2005	USA	Prospective study	1993–2000	Esophagus	1142	Before diagnosis	≥30 25–29.9	<25	0.84 (0.58, 1.19) 0.65 (0.5, 0.86)	0.78 (0.55, 1.12) 0.67 (0.51, 0.88)	Sex, stage, and income

Morgan et al., 2007	Wales	Prospective study	1995–2005	Esophagus	215	Before operation	≥25	≤25	NA	1.1 (0.73, 1.66)	Age, stage, and ASA grade

Healy et al., 2007	Ireland	Retrospective study	1998–2005	Esophagus	150	Before operation	≥30	<30	1.16 (0.66, 2.02)	NA	NA

Skipworth et al., 2009	UK	Prospective study	2001–2004	Esophagus	93	Before operation	>25	<25	0.78 (0.38, 1.6)	NA	NA

Madani et al., 2010	Canada	Prospective study	1991–2006	Esophagus	142	Before operation	≥30	<30	0.57 (0.38, 0.88)	0.57 (0.35, 0.93)	Age, sex, resection, grade, stage, and lymph node metastasis

Grotenhuis et al., 2010	Netherlands	Prospective study	1991–2007	Esophagus	556	Before operation	≥30	<18.5	0.58 (0.32, 1.04)	NA	NA

Hayashi et al., 2010	USA	Retrospective study	NA	Esophagus	301	Before treatment	≥25	<25	0.64 (0.44, 0.93)	0.62 (0.44, 0.88)	Age, weight loss, PVD, and stage

Melis et al., 2011	USA	Prospective study	1994–2010	Esophagus	490	Before operation	≥30	20–24	0.69 (0.51, 0.92)	NA	NA

Yoon et al., 2011	USA	Prospective study	1980–1997	Esophagus	778	Before operation	≥30	18.5–24.9	NA	1.01 (0.67, 2.23)	Age, sex, stage, grade, and weight loss

Scarpa et al., 2013	Italy	Retrospective study	2000–2008	Esophagus	278	Before diagnosis	>30	<20	NA	0.61 (0.4, 0.93)	Age, sex, stage, and weight loss

Blom et al., 2012	Netherlands	Prospective study	1993–2010	Esophagus	736	Before operation	≥30	<25	0.92 (0.8, 1.15)	NA	NA

Miao et al., 2015	China	Prospective study	2006–2012	Esophagus	1342	Before operation	≥25	18.5–24.9 <18.5	1.11 (0.89, 1.38) 1.48 (1.07, 2.04)	1.05 (0.84, 1.3) 1.16 (0.84, 1.6)	Age, sex, drinking, smoking, hypertension, diabetes, tumor length, differentiation, grade, stage, weight loss, and adjuvant chemoradiation

Minami et al., 2015	Japan	Prospective study	1997–2005	Stomach	1283	At diagnosis	≥25 18.5–23	23–25	NA	1.28 (0.93, 1.77) 1.5 (1.14, 1.98)	Age, sex, stage, histology, occupation, smoking, drinking, and family history

Ejaz et al., 2014	USA	Prospective study	2000–2012	Stomach	775	Before operation	≥30 25.0–29.9 <18.5	18.5–24.9	NA	1.13 (0.79, 1.61) 0.91 (0.66, 1.27) 1.5 (0.93, 2.41)	Age, race, preoperative albumin, chemotherapy, comorbidities, tumor size, type, morphology, T stage, AJCC stage, grade, lymph-vascular invasion, perineural invasion, and signet ring cell

Lee et al., 2015	Korea	Retrospective study	2000–2008	Stomach	1909	Before operation	≥25 <18.5	18.5–24.9	NA	0.64 (0.41, 1.02) 1.01 (0.72, 1.4)	Age, sex, surgery, tumor stage, histology, and curative resection

Liu et al., 2016	China	Prospective study	2004–2013	Stomach	320	Before operation	24–32.2	15.1–24	0.57 (0.37, 0.9)	0.31 (0.12, 0.8)	Age, sex, albumin, total cholesterol, triglyceride, high- and low-density lipoprotein cholesterol, cell differentiation, invasion depth, lymph node metastasis, distant metastasis, and stage

Meyerhardt et al., 2008	USA	Prospective study	1999–2001	Colorectum	1053	After operation	≥35 30–34.9 25–29.9	18.5–24.9	0.88 (0.58, 1.36) 0.93 (0.66, 1.31) 0.84 (0.61, 1.15)	0.87 (0.54, 1.42) 0.9 (0.61, 1.34) 0.72 (0.5, 1.03)	Age, sex, bowel wall invasion, lymph node metastasis, bowel perforation, obstruction, baseline performance status, treatment, weight loss, smoking, and activity level

Baade et al., 2011	Australia	Prospective study	2003-2004	Colorectum	1825	Before diagnosis	≥30 25–29.9 <18.5	18.5–24.9	NA	0.78 (0.59, 1.03) 0.75 (0.61, 0.94) 2.29 (1.47, 3.59)	Age, sex, physical activity, smoking status, marital status, education, insurance, tumor site, stage, treatment, and comorbidities

Campbell et al., 2012	USA	Prospective study	1992–2007	Colorectum	1957	Before diagnosis	≥30 25–29.9 <18.5	18.5–24.9	NA	0.93 (0.75, 1.17) 0.83 (0.7, 1) 1.3 (0.82, 2.06)	Age, sex, smoking, physical activity, red meat intake, SEER summary, and stage at diagnosis

Kuiper et al., 2012	USA	Prospective study	1993–1998	Colorectum	587	Before diagnosis	≥30 25–29.9	18.5–24.9	NA	1.09 (0.65, 1.83) 0.77 (0.47, 1.27)	Age at diagnosis, education, time from diagnosis to measurement, tumor stage, race, education, drinking, smoking, hormone replacement therapy, and prediagnostic physical activity

Li et al., 2009	USA	Retrospective study	2004–2008	Pancreas	609	At diagnosis	≥30 25–29.9	18.5–24.9	NA	1.86 (1.35, 2.56) 1.26 (0.94, 1.69)	Sex, race, diabetes, stage, tumor resection status, CA19-9 level, margin, and node status

Tsai et al., 2010	USA	Prospective study	1995–2005	Pancreas	795	Before operation	≥30 25–29.9	18.5–24.9	0.75 (0.58, 0.98) 0.73 (0.6, 0.88)	0.73 (0.56, 0.95) 0.74 (0.61, 0.89)	Age, sex, race, tumor differentiation and size, surgical details, perineural invasion, margin and node status, and weight loss

McWilliams et al., 2010	USA	Prospective study	2000–2009	Pancreas	1861	At diagnosis	≥30 25–29.9 <18.5	18.5–24.9	NA	1.25 (1.1, 1.41) 1.02 (0.89, 1.16) 1.42 (0.76, 2.68)	Age, sex, and diabetes status

Olson et al., 2010	USA	Retrospective study	2004–2008	Pancreas	314	At diagnosis or after treatment	≥30 25–29.9	18.5–24.9	1.17 (0.82, 1.68) 0.92 (0.89, 1.69)	1.38 (0.9, 2.11) 1.05 (0.7, 1.57)	Age, gender, smoking, diabetes, family history, chemotherapy, tumor stage, and history of allergies

Dandona et al., 2011	USA	Retrospective study	1995–2009	Pancreas	355	Before operation	≥30 25–29.9	18.5–24.9	0.85 (0.61, 1.2) 1.01 (0.76, 1.34)	NA	NA

Gong et al., 2012	USA	Retrospective study	1995–1999	Pancreas	510	At diagnosis	≥30 25–29.9	18.5–24.9	1.27 (0.93, 1.72) 1.01 (0.83, 1.22)	1.28 (0.91, 1.81) 1.04 (0.83, 1.28)	Age, sex, race, education level, smoking, and diabetes status

Gaujoux et al., 2012	USA	Prospective study	2000–2005	Pancreas	328	Before operation	≥30 25–29.9 <18.5	18.5–24.9	1.1 (0.8, 1.52) 1.21 (0.92, 1.6) 1.29 (0.67, 2.48)	NA	NA

Yuan et al., 2013	USA	Prospective study	1988–2010	Pancreas	902	Before diagnosis	≥30 25–29.9	18.5–24.9	NA	1.27 (1.03, 1.52) 0.94 (0.82, 1.07)	Age at diagnosis, sex, race, smoking, year, and stage at diagnosis

Pelucchi et al., 2014	Italy	Prospective study	1982–2007	Pancreas	644	At diagnosis	≥30 25–29.9	18.5–24.9	NA	1.32 (0.98, 1.79) 1.14 (0.94, 1.39)	Age and calendar period at diagnosis, study center, sex, and smoking

Kasenda et al., 2014	Switzerland	Retrospective study	1994–2004	Pancreas	483	At diagnosis	≥30 25–29.9 <18.5	18.5–24.9	2.02 (1.42, 2.89) 1.53 (1.22, 1.91) 1.06 (0.75, 1.46)	NA	NA

UV = univariate, MV = multivariate, HR= hazard ratio, RT = radiotherapy, ASA = American Society of Anesthesiology, NA = not available, PVD = peripheral vascular disease, SEER = surveillance, epidemiology, and END results.

**Table 2:** Random-effect summary estimates of the hazard ratios (HRs) of the association of OS of DSC except pancreatic cancer with highest versus lowest BMI comparison and site-specific analysis of digestive system cancers.

	Study	HR (95% CI)	*I*-squared	*P*_heterogeneity_
Region				
North America	11	0.77 (0.65, 0.89)	32.5%	0.139
Other regions	13	0.84 (0.70, 0.99)	72.2%	<0.001
Number of patients				
≤500	11	0.76 (0.55, 0.96)	81.4%	<0.001
>500	13	0.87 (0.76, 0.98)	33.5%	0.115
Study type				
Retrospective	8	0.77 (0.53, 1.01)	79.8%	<0.001
Prospective	16	0.84 (0.72, 0.95)	49.3%	0.014
Adjusted for covariates				
Yes	18	0.80 (0.68, 0.93)	54.2%	0.003
No	6	0.86 (0.70, 1.02)	67.7%	0.008
Adjusted for weight loss				
Yes	4	0.78 (0.52, 1.05)	58.0%	0.068
No	20	0.83 (0.71, 0.94)	68.3%	<0.001
Adjusted for tumor grade				
Yes	6	0.99 (0.70, 1.28)	51.9%	0.065
No	18	0.78 (0.67, 0.90)	74.2%	<0.001
Site-specific analysis of digestive system cancers				
Oropharynx	3	0.84 (0.37–1.32)	77.7%	0.011
Esophagus	12	0.77 (0.66–0.89)	35.7%	0.105
Stomach	4	0.82 (0.40–1.25)	81.6%	0.001
Colorectum	4	0.87 (0.73–1.01)	0.0%	0.685

HR = hazard ratio.

## Abbreviations

BMI: Body mass index
OS: Overall survival
DSC: Digestive system cancers
HRs: Hazard ratios
95% CIs: 95% confidence intervals
NOS: Newcastle Ottawa Scale.
